# Composition and Corrosivity of Extracellular Polymeric Substances from the Hydrocarbon-Degrading Sulfate-Reducing Bacterium *Desulfoglaeba alkanexedens* ALDC

**DOI:** 10.3390/microorganisms9091994

**Published:** 2021-09-21

**Authors:** Irene A. Davidova, Tiffany R. Lenhart, Mark A. Nanny, Joseph M. Suflita

**Affiliations:** 1 Department of Microbiology and Plant Biology, University of Oklahoma, Norman, OK 73019, USA; davidova@ou.edu (I.A.D.); tiffany-lenhart@ouhsc.edu (T.R.L.); 2 School of Civil Engineering and Environmental Science, University of Oklahoma, Norman, OK 73019, USA; nanny@ou.edu

**Keywords:** SRB, EPS, corrosion

## Abstract

Sulfate-reducing bacteria (SRB) often exist as cell aggregates and in biofilms surrounded by a matrix of extracellular polymeric substances (EPSs). The chemical composition of EPSs may facilitate hydrophobic substrate biodegradation and promote microbial influenced corrosion (MIC). Although EPSs from non-hydrocarbon-degrading SRB have been studied; the chemical composition of EPSs from hydrocarbon-degrading SRBs has not been reported. The isolated EPSs from the sulfate-reducing alkane-degrading bacterium *Desulfoglaeba alkanexedens* ALDC was characterized with scanning and fluorescent microscopy, nuclear magnetic resonance spectroscopy (NMR), and by colorimetric chemical assays. Specific fluorescent staining and ^1^H NMR spectroscopy revealed that the fundamental chemical structure of the EPS produced by *D*. *alkanexedens* is composed of pyranose polysaccharide and cyclopentanone in a 2:1 ratio. NMR analyses indicated that the pyranose ring structure is bonded by 1,4 connections with the cyclopentanone directly bonded to one pyranose ring. The presence of cyclopentanone presumably increases the hydrophobicity of the EPS that may facilitate the accessibility of hydrocarbon substrates to aggregating cells or cells in a biofilm. Weight loss and iron dissolution experiments demonstrated that the EPS did not contribute to the corrosivity of *D. alkanexedens* cells.

## 1. Introduction

Planktonic and biofilm-associated anaerobes, most notably sulfate-reducing bacteria (SRB), have long been implicated in metal biocorrosion processes [1,2]. It is known that their sulfidogenic and acidic metabolic products can cause oil souring, pipeline deterioration through metal loss, infrastructure damage, and sometimes unintended product releases [3]. Many microorganisms, including SRB, exist as cell aggregates, or biofilms, surrounded by a matrix of extracellular polymeric substances (EPSs). Among their many functions, EPSs provide cell protection, allow for biofilm stabilization and substratum attachments, and are a source of microbial nutrition [4,5,6]. The structural integrity of EPSs critically depends on the extracellular material produced by the biofilm-associated cells [5,7].

The EPSs from several terrestrial and marine bacteria have been characterized. The composition and physicochemical properties of a particular EPS can vary dramatically as a result of abiotic environmental factors (i.e., pH, temperature, salinity), nutritional conditions, and the individual microbial genetic makeup [6,7,8,9,10,11]. In general, extracellular material can account for more than 90% of the dry weight of a biofilm and may contain a combination of modified polysaccharides, proteins, nucleic acids, lipids, and bacterial metabolites, all of which determine the architecture of the matrix as well as its interaction with a substratum or surface [4,5,6,11].

Amphiphilic EPSs of several marine hydrocarbon-degrading bacteria have also been shown to be important for solubilizing and enhancing the bioavailability and biodegradation of hydrocarbon compounds, a characteristic that may be of potential biotechnological use in response to oil spills. For example, members of the genus *Halomonas* that degrade select aliphatic and polyaromatic hydrocarbons (PAHs) [12] produce EPSs that can solubilize PAHs and emulsify various hydrocarbon-containing compounds. This includes *n*-alkanes, food-grade oils, and crude oil, thus increasing the bioavailability of hydrophobic materials for biodegradation [12,13]. These capabilities are mostly attributed to relatively higher amounts of peptides and uronic acid in *Halomonas*-produced EPSs that account for their amphiphilic nature [8]. Interestingly, the presence of EPSs from *Halomonas* strain TG39 was shown to increase the biodegradation of phenanthrene by a Deepwater Horizon microbial community, suggesting that EPSs may help facilitate microbial access to potential carbon substrates in oil droplets [12]. 

The EPS of non-hydrocarbon-degrading SRB, most notably *Desulfovibrio* spp, has been studied. Exopolymers produced by two *Desulfovibrio* strains (Al1 and Ind1, later classified as *D. alaskensis* G20 [14] and *D. indonesiensis* [15], respectively) isolated from marine corrosion failures were determined to contain neutral hexoses, uronic acids, amino sugars, protein, and nucleic acids [16]. Additionally, the EPS of the *D. indonesiensis* Ind1 strain was also observed to contain an iron-chelating carbohydrate–protein complex that was corrosive to mild steel [17].

Thus, previous investigations have described and analyzed the composition, potential applications, and surface activity of EPSs produced from hydrocarbon-degrading marine bacteria and non-hydrocarbon-degrading SRB. However, there is a paucity of information on the function or composition of EPSs from anaerobic microbes growing on hydrophobic substrates. Indeed, “hydrophobic substrates” is far too broad a class of materials to consider in a single study. In this paper we have focused on the EPS produced by *Desulfoglaeba alkanexedens*, str. ALDC, an anerobic *n*-alkane-degrading sulfate-reducing bacterium [18] previously shown to be corrosive [19] that forms biofilm aggregates in pure cultures. Here we used *D. alkanexedens* as a model organism for studying the composition of EPSs derived from an anaerobic hydrocarbon-degrading bacterium and assessed the contribution of EPSs to corrosion in anoxic hydrocarbon-laden environments.

In this study, scanning electron microscopy (SEM), fluorescent microscopy, and nuclear magnetic resonance spectroscopy (NMR) were used to characterize the chemical composition of EPSs from decane-amended *D. alkanexedens* cultures. We determined that the fundamental chemical structure of the *D. alkanexedens* EPS is largely composed of a polysaccharide backbone with oxygenated alkyl functional groups bonded to the saccharide ring via carbon–carbon bonding. Although negligible amounts of proteins and lipids were detected, it must be noted that EPSs are typically a complex composition of biomolecules, several of which may be lost during EPS isolation and purification or present at concentrations below the detection limits of the utilized analytical methodology. The EPS characterized in this study is the bulk of the collected EPS material collected and presumably representative of the fundamental EPS chemical structure. The largely saccharide nature of EPSs was confirmed by liquid ^1^H NMR experiments. We also assessed the potential corrosivity of *D. alkanexedens* EPSs upon exposure to carbon steel coupons. Our results suggest that the EPS of this organism is not particularly corrosive and may protect the metal from oxidation. The chemical composition of this EPS suggests that the *D. alkanexedens* biofilm matrix is less important for carbon steel corrosion than the metabolic end products of hydrocarbon metabolism but may facilitate microbial access to hydrocarbon substrates at an oil–water interface.

## 2. Materials and Methods

### 2.1. Incubations

*Desulfoglaeba alkanexedens* str. ALDC previously isolated from sludge collected from a navy oil waste facility (Portsmouth, VA, USA) was cultivated under anaerobic sulfate-reducing conditions as previously described [18]. Briefly, *D. alkanexedens* was incubated in a basal reduced seawater medium [20] supplemented with a 0.001% yeast extract (BD Bioscinces, Miami, FL, USA) and 10 mM decane. The cultures were incubated at 31 °C in hermetically closed serum bottles with a headspace containing N_2_/CO_2_ (80:20%).

### 2.2. EPS Extraction

*D. alkanexedens* aggregates were collected and subjected to low-speed centrifugation (3000× *g* for 15 min, Beckman J2-21, Palo Alto, CA, USA). The aggregates were washed with phosphate buffered saline (PBS) (pH 7.2) and resuspended to 1/10 of the original volume. The aggregates were then vortexed for 5 min and the cells were removed by centrifugation (11,000× *g* for 10 min). The resulting supernatant was subject to ultracentrifugation (30,000× *g* for 40 min) to remove the membrane debris followed by an overnight dialysis (2 KDa cut-off) against nanopure water.

### 2.3. Field Emission Scanning Electron Microscopy (FE-SEM)

The cell aggregates were pipetted onto poly-L-lysine (0.1% *w*/*v* solution, Sigma, St. Louis, MO, USA)-coated cover slips and incubated at 20 °C for 1 h. The affixed aggregates were gently rinsed 2 X in sterile water followed by immersion in a mixture of 2.5% glutaraldehyde (Sigma-Aldrich, St. Luis, MO, USA) and a 0.1 M sodium cacodylate buffer and incubated at 4 °C overnight. The fixed cell aggregates were washed in 0.1 M of a cacodylate buffer (3 × 10 min) to remove the excess fixative. Post-fixation, the samples were immersed in 1 mL of an OsO_4_ buffer for 1 h at 4 °C followed by 3 washes in distilled water. The fixed aggregates were dehydrated by immersion in a graded ethanol series (one wash each at 25%, 50%, 70%, 85%, 95% ethanol, and finally 3 washes at 100% ethanol). The final dehydration was performed by 3 × 10 min washes in hexamethyldisilazane followed by air drying. The glass coverslips with the affixed dehydrated cell aggregates were sputter-coated with AuPd prior to imaging. The prepared samples were imaged with Zeiss NEON FE-SEM (Carl Zeiss Microscopy, Jena, Germany) at a 3–5 mm working distance and 1.0 kV electron beam.

### 2.4. Confocal Microscopy

The cell aggregates were affixed to poly-L-lysine-coated glass slides as described above. The attached biofilms were fixed with 1% paraformaldehyde/PBS (Sigma-Aldrich, St. Luis, MO, USA) for 30 min followed by a PBS wash to remove the fixative. The fixed biofilms were stained with 100 μg/mL concanavalin A tetramethyl rhodamine, a carbohydrate-binding fluorophore stain, and 10 μM of a Syto^TM^ 9 green fluorescent nucleic acid stain for 30 min. The samples were gently rinsed to remove the excess dye and a coverslip was secured on top of the stained samples. The imaging was performed on a Leica SP8 scanning confocal microscope (Leica Microsystems GmbH, Wetzlar, Germany) using 488 nm argon laser filters for Syto^TM^ 9 and a 561 nm diode-pumped solid state laser for rhodamine.

### 2.5. Nuclear Magnetic Resonance (NMR) Analysis

The EPS samples were dissolved in deuterium oxide. ^1^H NMR spectra were collected on a 500 MHz NMR spectrometer (Varian (Agilent), Palo Alto, CA, USA) using 1d_TNNOESY (relaxation delay = 1 s, *n* = 512) and a Gradient-COSY with a non-uniform sampling technique (relaxation delay = 1 s, number of increments in the indirect dimension = 256, *n* = 16). The experiments included ‘presat’ water suppression.

### 2.6. Corrosion Analysis

The isolated EPSs were incubated in an anaerobic chamber with mild carbon steel standard grade 10180 coupons according to the unified numbering system for metals and alloys (UNS) described in [21]. Aliquots (2 mL) of the extracted EPS, live *D. alkanexedens* culture, or filter-sterilized live culture (filtrate) were pipetted into a 24-well plastic tissue culture dish in an anaerobic chamber. Pre-cleaned (acetone-rinsed and N_2_-dried), sterile, pre-weighed steel coupons were transferred into each well and the samples were covered and incubated under anaerobic conditions for 6 weeks. Initial (day 0) and final (day 41) analyses included coupon weight loss and a total iron analysis using a ferrozine reagent [22]. The final weight loss was translated into corrosion rates (milli-inch per year; 1 mpy = 0.0254 mm/year) according to ASTM Standard G1-03 [23].

### 2.7. Polysaccharide Characterization

Neutral sugars (hexoses and pentoses), hexosamines, hexuronic acids, and deoxyhexoses were analyzed by colorimetric techniques described in [24].

## 3. Results

### 3.1. D. alkanexedens Aggregation and Extracellular Material

**Figure 1:**
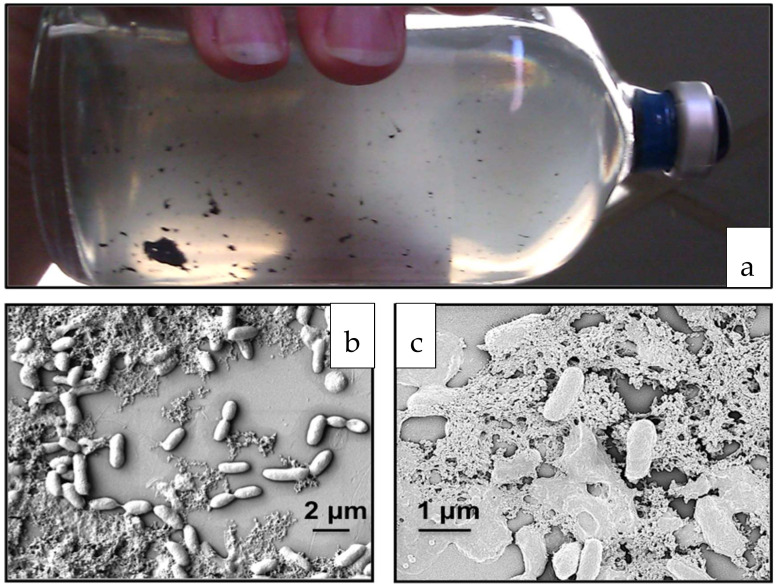
*Desulfoglaeba alkanexedens* str. ALDC forms aggregates and produces extracellular material: (**a**) cell aggregates formed by *n*-decane-amended incubations; (**b**) and (**c**) scanning electron micrographs of the biofilms surrounded by extracellular matrix.

It was previously observed that aggregates of *Desulfoglaeba alkanexedens* formed when the organism was cultivated in an aqueous medium with *n*-alkanes as electron donors and sulfate as an electron acceptor [17]. As illustrated in Figure 1a, *D. alkanexedens* utilizing *n*-decane as a substrate formed irregularly shaped aggregates. The black colored (presumably iron sulfide-laden) aggregates varied in size from < 1 mm to > 10 mm. When individual clumps were imaged at a high magnification by FE-SEM, biofilms containing *D. alkanexedens* cells surrounded by and imbedded in extracellular material could easily be observed (Figure 1b,c).

### 3.2. Fluorescent Microscopic Analysis of D. alkanexedens Aggregates

**Figure 2:**
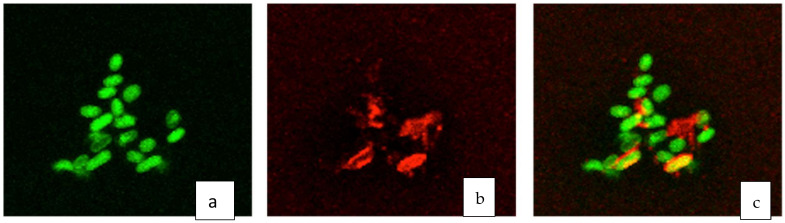
Confocal images of *D. alkanexedens* EPS with fluorophore stains: (**a**) nucleic acid stain Syto 9^TM^; (**b**) carbohydrate-binding conconavalin A and (**c**) overlayed image. Bacterial cells are 1–2 microns in size.

Pursuant to the observation that *D. alkanexedens* cells formed biofilm aggregates, we sought to determine the nature of the presumed EPS. As most bacterial EPS matrices are composed of polysaccharides, we attached *D. alkanexedens* biofilm aggregates to glass slides and stained the preparations with concanavalin A and the nucleic acid stain Syto-9. Fluorescent microscopic images illustrate the *D. alkanexedens* cells stained with the nucleic acid fluorophore (Figure 2a) and the EPS stained with the carbohydrate-binding concanavalin A fluorophore (Figure 2b). The overlayed images (Figure 2c) clearly show the individual *D. alkanexedens* cells (green) within the biofilm surrounded by extracellular regions containing the concanavalin A carbohydrate stain (red). Areas where the nucleic acid and the carbohydrate stains overlapped are shown as yellow. By this analysis, it seems clear that the carbohydrate was present immediately surrounding the cells or within the biofilm, suggesting that it was produced by the cells and likely involved in biofilm aggregation.

### 3.3. EPS Composition and Structure

Once it was determined from the SEM and fluorescent microscopic analyses that *D. alkanexedens* cells were imbedded and surrounded by EPSs composed, at least in part, of carbohydrates, we attempted to determine the chemical composition of the polymeric material. The colorimetric biochemical analyses used to determine the various classes of carbohydrate contained in the EPSs (including assays for neutral sugars (hexoses and pentoses), hexosamines, hexuronic acids, and deoxyhexoses) were generally unsuccessful with the *D. alkanexedens* EPS sample, resulting in only microgram amounts of neutral sugars and no evidence for the presence of any other carbohydrate constituent. The EPS yield from *D. alkanexedens* was persistently low (<1 g material/L culture) and contained too little material for definitive results with bench top methods. With this in mind, we utilized proton NMR as an improved method to obtain the structural information associated with *D. alkanexedens* EPSs.

### 3.4. Nuclear Magnetic Resonance Analysis

**Figure 3:**
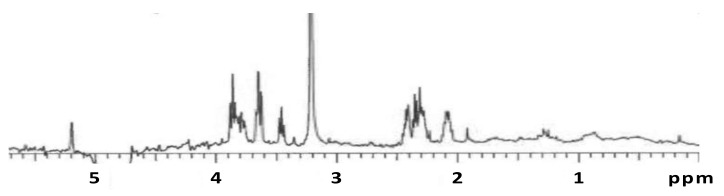
^1^H NMR spectrum of EPS isolated from *D. alkanexedens*.

The ^1^H-NMR spectrum (Figure 3) showed three peak clusters between δ 3.40 and 4.00 indicative of the protons in a pyranose saccharide with the proton attached to the anomeric carbon appearing as a single peak at δ 5.19 [24]. Three additional peak clusters were present between δ 2.00 and 2.50 indicating the protons in acetyl groups or α-carbons of carbonyl groups [25,26]. The two sharp peaks at δ 1.91 and 3.24 were from acetate and methanol, respectively, resulting from the microbial media and buffer.

**Figure 4:**
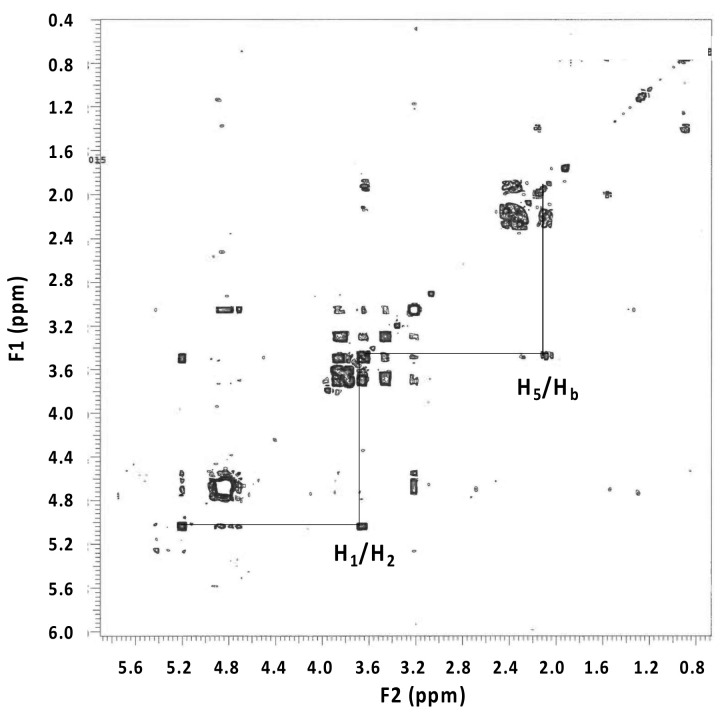
Gradient COSY spectrum illustrating the coupling between the proton attached to the anomeric carbon and the adjacent proton on the pyranose ring as well as the coupling between the pyranose ring proton at d 3.62 and the proton at d 2.10.

The ratio of the integrated peak areas was 1:53 for the pyranose protons, including the anomeric proton, to the protons between δ 2.00 and 2.50. The gradient COSY spectrum (Figure 4) showed coupling between the proton attached to the anomeric carbon and the adjacent proton on the pyranose ring as well as coupling between the pyranose ring proton at δ 3.62 and the proton at δ 2.10. Multiple couplings between all the protons appeared between δ 2.00 and 2.50 illustrating that the chemical structure was cyclic rather than linear.

The elucidation of the chemical structure of the EPS repeating saccharide unit assumed that the H_6_ proton of the pyranose ring was part of the cyclic chemical structure appearing between δ 2.00 and 2.50. Using the ratio of the integrated peak areas of 1:53, a disaccharide pyranose system with 12 protons resulted in the attached cyclic chemical structure having 7 to 8 protons.

**Figure 5:**
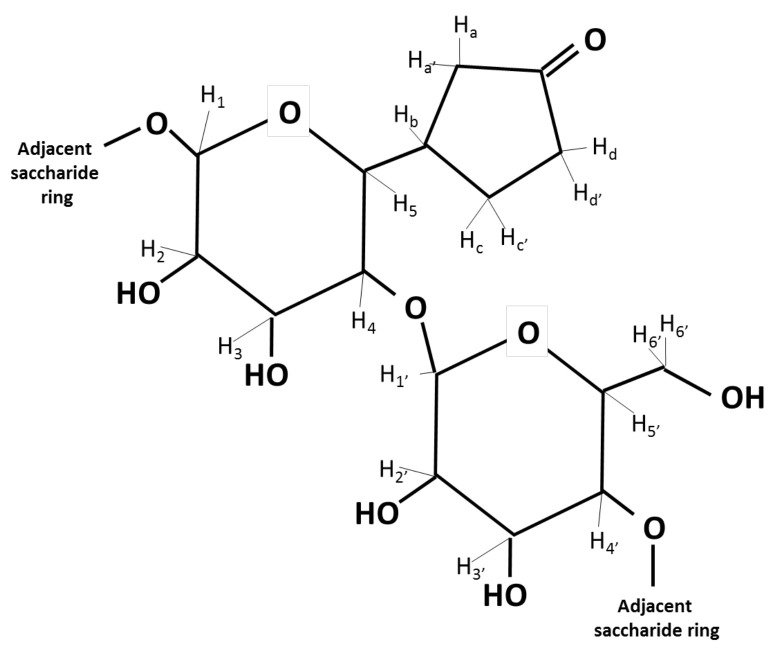
EPS chemical structure consists of two pyranose rings and a cyclopentanone moiety.

Using ^1^H NMR chemical shift prediction software (nmrdb.org), the optimal chemical structure consisted of two pyranose rings and a cyclopentanone moiety (Figure 5). The slight discrepancy between the experimental and the predicted ^1^H chemical shift values for the pyranose rings was likely because the stereochemistry was not accounted for in the prediction software (Table 1). However, a close alignment existed between the EPS experimental ^1^H chemical shift values and those of starch in D_2_O [27], supporting a disaccharide repeating unit for the EPS.

### 3.5. Assessment of D. alkanexedens EPS Corrosivity

**Figure 6:**
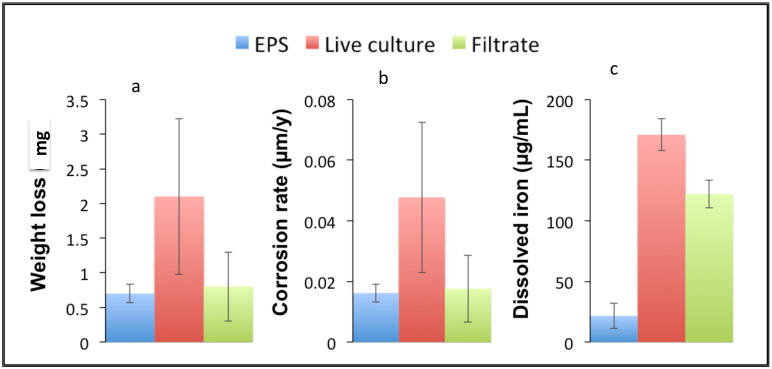
EPS corrosivity relative to the live cells and cultural fluid: (**a**) coupon weight loss; (**b**) corrosion rate based on the weight loss; (**c**) dissolved total iron.

Based on the determination that *D. alkanexedens* forms biofilm aggregates and produces EPSs, we sought to determine if this specific EPS directly contributed to the corrosivity of the organism. To that end, we compared isolated EPSs with the same volume of either a live *D. alkanexedens* culture or a cell-free filter-sterilized spent culture fluid. These preparations were incubated in contact with pre-weighed carbon steel coupons under anaerobic conditions for approximately 6 weeks. At the end of the experiment, the coupon weight loss and total dissolved iron release were assessed. The coupons incubated with EPSs (Figure 6, blue bar) experienced less than 1 mg weight loss compared with a 2 mg weight loss when incubated with the live culture (Figure 6, red bar). Although both conditions showed a minimal weight loss, the EPSs clearly did not contribute to metal loss from the coupon as the total coupon weight loss was similar to that of the filter-sterilized preparation (Figure 6, green bar). Similarly, when a corrosion rate was calculated (Figure 6b), the *D. alkanexedens* EPS showed a corrosion rate that was approximately 2 × lower than that of the live culture (0.016 versus 0.047 μm/y, respectively). The dissolved iron measurements as another measure of corrosion (Figure 6c) exhibited a comparable trend with an increased iron dissolution in the live culture-amended sample versus the EPS-amended sample. In this assessment, the iron dissolution in the live culture-amended preparations was dramatically higher than that of the EPS-amended incubations (171 versus 21 μg/mL, respectively). However, the observation that the total iron concentration was significantly lower than both the live culture and filter-sterilized samples argues that the *D. alkanexedens* EPS was moderately protective.

## 4. Discussion

The sulfate-reducing delta-proteobacterium *Desulfoglaeba alkanexedens*, which oxidizes *n*-alkanes under anaerobic conditions, tends to exist in aggregates in a liquid medium or as biofilms on solid surfaces. The same flocculated growth pattern has been observed for other microbes growing on hydrocarbons [28]. Obviously, such a lifestyle is advantageous for their development. Most probably, in the case of hydrocarbon-degrading bacteria, the aggregated growth is influenced by the hydrophobicity of the substrates. Alkane uptake requires a bi-phasic system (an alkane droplet/solid and a microbial cell) coming into contact, resulting in the transfer of a hydrocarbon molecule to the cell. Although alkane transport is not well-understood in anaerobes, studies have been performed on aerobic alkane-degrading bacteria.

It was proposed that alkanes could be transported into a cell by direct contact via diffusion or active transport [29,30]. Another hypothesis was that alkanes passed through hydrophobic compartments in the cell wall [31]. To this end, cells can modify their membrane composition and structure; for instance, by releasing outer membrane vesicles [32] so that the cells become more hydrophobic.

The EPS produced by anaerobic microbes can vary its hydrophilicity or hydrophobicity depending upon its chemical composition. The EPS extracted from anaerobic granular sludge produced in wastewater reactors is chemically similar to alginate that contains carboxylate functional groups [33] whereas the EPS from anaerobic anammox bacteria is less polar and prone to aggregation due to aldehyde and aromatic functional groups incorporated with a polysaccharide backbone [34]. As the EPS produced by *D. alkanexedens* had a cyclopentane group linked to a pyranose ring, it was hypothesized that this structure indicated an increased hydrophobicity. This hydrophobicity likely resulted in the partitioning of an alkane droplet into the EPS, thereby facilitating its uptake by the anaerobic bacterium. Thus, among other important functions, EPSs are likely to be important for the bioavailability of hydrophobic substrates; in this particular case, *n*-alkanes.

Previous research has illustrated that bacterial EPSs can be either corrosive or passivating, depending on their chemical composition and the prevailing environmental conditions [35,36,37]. Experimental evidence from recent electrochemical and SEM studies indicated that *D. alkanexedens* is corrosive and propagates localized pitting on steel surfaces [19]. However, our experimentation suggests that EPSs did not contribute substantively to metal corrosion. Based on coupon weight loss, the corrosion by live cells was two times greater than that associated with the isolated EPS preparations and on a par with the sterile controls. The corrosion assessment based on the iron dissolution demonstrated dramatically higher increases in live cell preparations relative to the pure EPS preparation. The iron dissolution by the pure EPS preparations was even less than that measured in the filter-sterilized controls, arguing that EPSs produced by *D. alkanexedens* may be somewhat protective. The lack of corrosivity supported an EPS chemical structure containing a cyclopentanone that would not covalently bond with oxidized metal ions.

## 5. Conclusions

The hydrocarbon-degrading sulfate-reducing bacterium *Desulfoglaeba alkanexedens* str. ALDC aggregated and formed biofilms both in a solution and on surfaces. Specific fluorescent staining and ^1^H NMR spectroscopy revealed that the fundamental chemical structure of *D. alkanexedens* EPSs was composed of a pyranose polysaccharide and cyclopentanone in a 2:1 ratio. A 2D COSY and a 1H NMR analysis indicated that the pyranose ring structure was bonded by 1,4 connections with the cyclopentanone directly bonded to one pyranose ring. The presence of cyclopentanone increased the hydrophobicity of the EPS, which might help facilitate the accessibility of the hydrocarbon substrates to aggregating cells or cells in a biofilm. Weight loss and total iron analyses indicated that the *D. alkanexedens* EPS was not corrosive and may be somewhat protective when compared with the cell-free culture fluids. This study is an initial characterization of EPSs from an anaerobic hydrocarbon-degrading bacterium and suggests that the biofilm matrix produced by this organism is less important for carbon steel corrosion than the metabolic products of hydrocarbon metabolism.

## Figures and Tables

**Table 1:** Proton chemical shift assignments for the experimental ^1^H NMR data, predicted ^1^H NMR data (nmrdb.org), and starch dissolved in D_2_O as a reference [26].

^1^H NMR Chemical Shift Values (ppm)			
	Experimental NMR	Predicted NMR	Starch in D_2_O
H_1_	5.19	4.58	5.35
H_2_	3.66	3.24	3.63
H_3_	3.87	3.65	3.94
H_4_	3.47	3.49	3.62
H_5_	3.62	3.52	3.82
H_b_	2.10	2.32	
H_a_ H_a′_	2.36	2.35, 2.45	
H_d_ H_d′_	2.42	2.42, 2.35	
H_c_ H_c′_	2.34	2.48, 2.27	
H_1′_	5.19	4.49	5.35
H_2′_	3.66	3.20	3.63
H_3′_	3.62	3.60	3.94
H_4′_	3.47	3.16	3.62
H_5′_	3.87	3.46	3.82
H_6′_ H_6′_	3.76	3.81, 3.81	3.87

## Data Availability

Not applicable.
